# Studies on the Small Body Size Mouse Developed by Mutagen *N*-Ethyl-*N*-nitrosourea

**DOI:** 10.5487/TR.2008.24.1.069

**Published:** 2008-03-01

**Authors:** QianKun Zhang, Kyu-Hyuk Cho, Jae-Woo Cho, Dal-Sun Cha, Han-Jin Park, Seokjoo Yoon, ShouFa Zhang, Chang-Woo Song

**Affiliations:** 110 Department of Research & Development, Korea Institute of Toxicology, Korea Research Institute of Chemical Technology, P.O BOX 123, Yuseong, Daejeon, 305-343 Korea; 210 Department of Veterinary Medicine, YanBian University, Longjing, China

**Keywords:** Small body size mice, Linkage analysis, Body weight, Bone mineral density

## Abstract

Mutant mouse which show dwarfism has been developed by *N*-ethyl-*N*-nitrosourea (ENU) mutagenesis using BALB/c mice. The mutant mouse was inherited as autosomal recessive trait and named Small Body Size (SBS) mouse. The phenotype of SBS mouse was not apparent at birth, but it was possible to distinguish mutant phenotype from normal mice 1 week after birth. In this study, we examined body weight changes and bone mineral density (BMD), and we also carried out genetic linkage analysis to map the causative gene(s) of SBS mouse. Body weight changes were observed from birth to 14 weeks of age in both affected (n = 30) and normal mice (n = 24). BMD was examined in each five SBS and normal mice between 3 and 6 weeks of age, respectively. For the linkage analysis, we produced backcross progeny [(SBS × C57BL/6J) F_1_ × SBS] N_2_ mice (n = 142), and seventy-four microsatellite markers were used for primary linkage analysis. Body weight of affected mice was consistently lower than that of the normal mice, and was 43.7% less than that of normal mice at 3 weeks of age (*P* < 0.001). As compared with normal mice at 3 and 6 weeks of age, BMD of the SBS mice was significantly low. The results showed 15.5% and 14.1% lower in total body BMD, 15.3% and 8.7% lower in forearm BMD, and 29.7% and 20.1% lower in femur BMD, respectively. The causative gene was mapped on chromosome 10. The map order and the distance between markers were *D10Mit248* - 2.1 cM - *D10Mit51* - 4.2 cM - ***sbs*** - 0.7 cM - *D10Mit283* - 1.4 cM - *D10Mit106* - 11.2 cM - *D10Mit170*.

## INTRODUCTION

With the completion of human genome project, mouse and rat genome sequence, systematic determination and analysis of gene function in mammalian genome are mainstream of the major scientific challenges for the 21st century (Lander *et al.*, 2001; Waterston *et al.*, 2002; Gibbs *et al.*, 2004). Mouse has been considered as a primary model for human disease research due to the similarity of its genome, developmental and biochemical pathways, and physiology to human (Soewarto *et al.*, 2000; Brown and Hardisty, 2003). Mouse mutagenesis is a powerful tool for study of mammalian gene function (Justice *et al.*, 1999). Currently mouse mutations have been generated in many laboratories by chemical mutagenesis (Davis *et al.*, 1999).

*N*-Ethyl-*N*-nitrosourea (ENU) is a potent chemical mutagen, which acts through random alkylation of nucleic acids, and it can randomly cause single base pair mutations in a wide variety of organisms (Russel *et al.*, 1979, 1982a, b; Hitotsumachi *et al.*, 1985; Justice and Bode 1986). The risk assessment of carcinogens and teratogenes are well studied but relatively mutagens like ENU are not (Purchase, 2001; Kwack and Cho, 2005; Kim *et al.*, 2006). In the mouse testis, the action of ENU is the most potent in spermatogonial stem cells, and it produces the highest mutation rate of any germline mutagen tested in the mouse with its optimal dose producing about one mutation per gene in every 175 to 655 gametes (Hitotsumachi *et al.*, 1985; Shelovsky *et*

*al.*, 1993; Justice *et al.*, 2000; Noveroske *et al.*, 2000). However low mutation frequencies of ENU-treated female mice have been reported (Ressell and Russell, 1992). Effective ENU doses and specific mutagenesis protocols have been optimized for various strains of mice to affect single gene mutations in the progeny of males exposed to the chemical. The mutant mice that are being created from ongoing ENU mutagenesis projects are an invaluable resource for the biomedical community. Further it may allow phenotype-driven approaches to isolate mutations in any gene of interest for short time to analyze gene function (Noveroske *et al.*, 2000).

Growth is an inherent property of life. Normal somatic growth requires the integrated function of many of the hormonal, metabolic, and other growth factors. The genetic etiology of growth insufficiency is very complicated. Dwarfism can be associated with a variety of complication and genetic heterogeneity, such as achondroplasia (Wang *et al.*, 1999; Argentin and Cicchetti, 2000), osteoporosis (Eason *et al.*, 1995), growth hormone, and many different factors (Lira *et al.*, 1993; Shibayama *et al.*, 1993; Zhou *et al.*, 1995). Mutations of genes involved in the process cause several types of dwarfism in human and mouse. Many mouse models associated with dwarfism have been known, such as Snell dwarf mouse, Ames dwarf mouse, SMA-1 mouse and achondroplastic mouse, etc. Four naturally occurring murine models of congenital and autosomal recessive GH deficiency exist: the Snell (*dw*), the Ames (*df*), the Spontaneous Dwarf Rat (*SDR*), and the little (*lt*) mouse (Jansson *et al.*, 1986; Godfrey *et al.*, 1993; Lin *et al.*, 1993; Li *et al.*, 1990; Sornson *et al.*, 1996; Takeuchi *et al.*, 1990). The Snell mouse results from a point mutation in the gene encoding for the pituitary transcription factor *Pit-1*, which is required for the development of all three pituitary cell lines (Li *et al.*, 1990). The Ames mouse results from a mutation of another transcription factor (*Prop-1*), which acts as activating factor of the *Pit-1* gene (Sornson *et al.*, 1996). The *SDR* rat has a point mutation in the *GH* gene (Takeuchi *et al.*, 1990). The little mouse has a missense mutation in the extracellular domain of the *GHRHR* gene, which does not allow proper binding of *GHRH* (Godfrey *et al.*, 1993; Lin *et al.*, 1993). The SMA-1 mouse is a novel ENU-induced mutant mouse, which is characterized by dwarfism (Meyer *et al.*, 2004). The achondroplastic mouse is a spontaneous mutant with an autosomal recessive gene (*cn*) which is characterized by disproportionate dwarfism due to disturb chondrogenesis during endochondral ossification (Tsuji and Kunieda, 2005).

In previous studies from our laboratory, we have reported generation of ENU-induced mutant mice that showed dwarfism (Cho *et al.*, 2003). The mutation was generated and identified in a large-scale ENU-mouse-mutagenesis program (Soewarto *et al.*, 2000; Hrabe de Angelis *et al.*, 2000). This program represents a powerful phenotype-driven approach to gene-function analysis and increases existing mouse mutant resources.

In this study, we examine body weight changes for further elucidating phenotypic character, and examine the bone mineral density (BMD) for exploring correlation between BMD and dwarfism phenotype, and map the causative gene for cloning of causative gene in the SBS mouse.

## MATERIALS AND METHODS

***Animals & environmental conditions.*** For breeding, one male mouse was usually maintained with two female mice. Male mice were separated from female mice as soon as pregnancy was assessed from weight gain in female mice or 2 weeks after breeding pairs. Breeding cages were observed for newborns every day, and gestation duration was calculated from the time interval between first day of breeding pair and resulting offspring day at birth. Offspring were generally weaned at 21 days, and individually marked, and housed in groups of individuals of the same sex. All mice have been maintained in the barrier system under the Specific-Pathogen Free (SPF) condition with regulated lighting (07:00~19:00 hour), temperature (23 ± 3°C), relative humidity (50 ± 10%), and air ventilation (10~12 times per hour). Pelleted food for experimental animals was purchased from Jeil Food (Daejeon, Korea), which was irradiated by gamma-ray at 2.0 Mrad (Greenpia, Korea) and given to mice *ad libitum*. Tap water was given to mice *ad libitum*, following the UV-irradiation and filtration. All animal experiments were carried out in accordance with *Guidelines for Animal Experimentation* and *Institutional Animal Care and Use.*

***Phenotype characterization and body weight changes.*** Phenotype characterization was examined from birth to 6 weeks of age (Fig. 1). Body weight was measured once a day from birth to 6 days of age and once a week from 1 to 14 weeks of age.

***BMD measurement.*** Bone mineral density (BMD) was examined at 3- and 6-week old SBS and normal mice for area for forearm, femur and total body. All mice to be examined were anaesthetized by intraperitoneal injection with Ketamine and Xylazine (0.05 mg of Ketamine and 0.015 mg of Xylazine per 1 g of body weight),

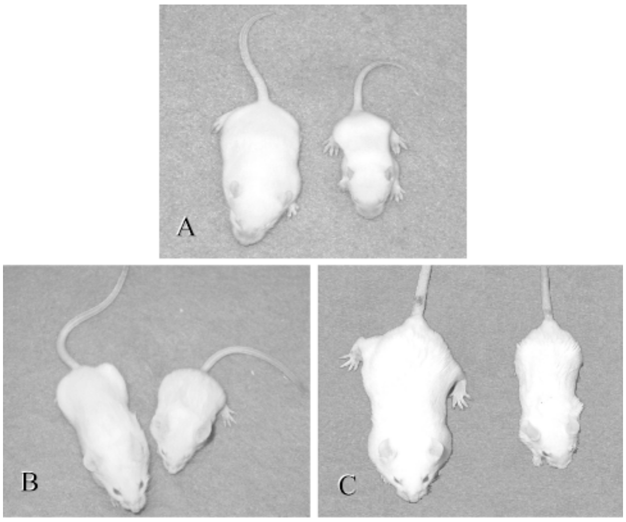

**Fig. 1.** Photographs of phenotype in the SBS mouse (A, B and C, left: normal mouse, right: SBS mouse, A; at 1 week of age, B; at 3 weeks of age, C; at 6 weeks of age).

Measurement were determined by the PIXImus small animal dual-energy X-ray absorptionmetry (DEXA) system (Lunar Corporation, USA). BMD is a two-dimensional measurement comprised of mineral with the area determined to be bone by the present thresholds in the PIXImus densitometer. Calibrations were performed with a phantom of known density, and quality assurance measurements were performed prior to BMD measurement.

***Genetic mapping.***

***Reagents and chemicals:*** In this study, chemicals were purchased from Sigma-Aldrich® (CA, USA) and Merck. Some products of Qiagen® (CA, USA) and Bioneer® (Cheong-won, Korea) were used in the molecular biology research.

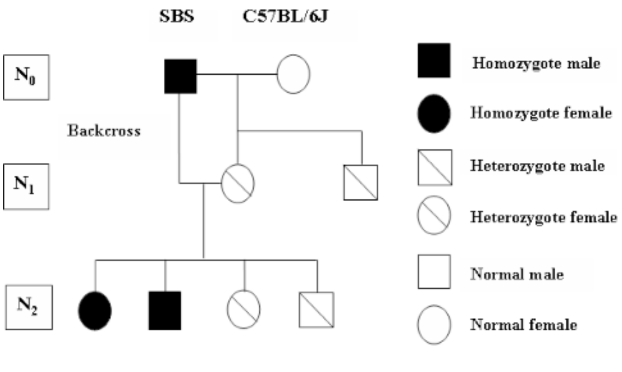

**Fig. 2.** Mating test for linkage analysis in SBS mice, Homozygote male mice were mated with C57BL/6J. Squares and circles denote male and female mice, respectively, filled symbols denote affected mice, half obliqued symbols denote heterozygote mice, and blank symbols denote normal mice.

***Microsatellite makers:*** Information of microsatellite markers had typed polymorphism between BALB/c and C57BL/6J was obtained form the MGI (www.informatics.jax.org). Microsatellite markers were purchased from Bioneer Inc. (Cheong-won, Korea).

***Mating experiment:*** SBS male mice were mated with female C57BL/6J mice according to 1 : 2 to produce (SBS × C57BL/6J) $F_1$ hybrids. Further, female mice of $F_1$ progeny were mated with parental SBS male mice by backcross to produce [(SBS × C57BL/6J) $F_1$ × SBS] $N_2$ progeny for linkage analysis (Fig. 2).

***Genotyping of simple sequence length polymorphisms (SSLPs).***

***DNA extraction:*** Genomic DNA was extracted from the mice tails by DNneasy tissue Kit (Qiagen, CA, USA) according to the manufacturer's protocols. Concentration and quality of genomic DNA were measured by Nanodrop 1000 (Nanodrop, DE, USA).

***Polymerase chain reaction:*** Polymerase chain reaction (PCR) amplifications were carried out in 20 μl reaction mixture containing 20 ng template DNA, 20 pmole primers and PCR premixture (Taq DNA polymerase 1 U, DNTP 250 μM, Tris-HCl 10 mM, KCl 40 mM, $MgCl_2$ 1.5 mM, Stabilizer and tracking dye) by Model T-Gradient (Biometra, Goettingen, Germany) and PTC-100 (MJ Research, NV, USA). PCR conditions consisted of one cycle of denaturation for 5 minutes at 94°C followed by 30 cycles with each cycle consisting of denaturation for 40 seconds at 94°C, annealing for 1 minute at 53~59°C regulated by different microstellite markers and extension for 1 minute at 72°C, and final extension 72°C for 10 minutes. The PCR products were stored at 4°C.

***Electrophoresis:*** The PCR products were electrophoresed in denaturing 8%~10% polyacrylamide gel in 0.5 × TBE buffer for 150~300 minutes under 120~150 Volts (Hu and O'shaughnessy 2001).

***Staining:*** The polyacrylamide gel with PCR products was stained in ethidium bromide (Et-Br) for 25~30 minutes and the DNA bands were visualized.

***Image analysis:*** The polyacrylamide gel with PCR products was imaged by ultraviolet ray in AlpaImager™ 3400 (Alpha innotech, CA, USA) and DNA bands were analyzed by AlphaEaseFC™ system program (Alpha innotech, CA, USA).

***Linkage analysis:*** Microsatellite markers were used for linkage analysis. We used total 79 microsatellite markers for linkage analysis; 75 markers for primary linkage screening and 4 markers for further mapping.

**Table 1.** Phenotype segregation of $N_2$ progeny analyzed in this study

| Mating | Phenotype | | | Segregation ratio | *P* value |
|---|---|---|---|---|---|
| | Total No. mice | No. affected mice | No. normal mice | | |
| Backcross | 142 (74/68)[a] | 72 (32/40)[a] | 70 (42/28)[a] | 1 : 1 | *P* > 0.05 |

[a](females/males).
Breeding data were analyzed by $X^2$-test and considerably significant at *P* > 0.05.

**Table 2.** Secondary screen using the thirty-eight microsatellite markers in the primary linkage analysis

| Chr.[a] | Markers | Position (cM) | Affected | | Normal | | Recombination value (%) | $X^2$ analysis |
|---|---|---|---|---|---|---|---|---|
| | | | Homo[b] | Hetero[c] | Homo[c] | Hetero[b] | | |
| **1** | ***D1Mit302*** | **32.8** | **13** | **9** | **7** | **15** | **36.4 ± 7.3** | **3.273** |
| 1 | *D1Mit33* | 81.6 | 8 | 14 | 10 | 12 | 54.5 ± 7.5 | 0.364 |
| 2 | *D2Mit329* | 49.2 | 12 | 10 | 11 | 11 | 47.7 ± 7.5 | 0.091 |
| 2 | *D2Mit285* | 86 | 14 | 8 | 12 | 10 | 45.5 ± 7.5 | 0.364 |
| 3 | *D3Mit22* | 33.7 | 14 | 8 | 12 | 10 | 45.5 ± 7.5 | 0.364 |
| 3 | *D3Mit19* | 61.8 | 13 | 9 | 14 | 8 | 52.3 ± 7.5 | 0.091 |
| 4 | *D4Mit178* | 35.5 | 15 | 7 | 11 | 11 | 40.9 ± 7.4 | 1.455 |
| **4** | ***D4Mit204*** | **61.9** | **12** | **10** | **7** | **15** | **38.6 ± 7.3** | **2.273** |
| **5** | ***D5Mit81*** | **28** | **14** | **8** | **7** | **15** | **34.1 ± 7.1** | **4.455** |
| 5 | *D5Mit210* | 64 | 9 | 13 | 12 | 10 | 56.8 ± 7.5 | 0.818 |
| 6 | *D6Mit8* | 35.2 | 8 | 14 | 9 | 13 | 52.3 ± 7.5 | 0.091 |
| 6 | *D6Mit254* | 60.55 | 10 | 12 | 14 | 8 | 59.1 ± 7.4 | 1.455 |
| 7 | *D7Mit176* | 27.0 | 14 | 8 | 9 | 13 | 38.6 ± 7.3 | 2.273 |
| **7** | ***D7Mit220*** | **52.4** | **15** | **7** | **8** | **14** | **34.1 ± 7.1** | **4.455** |
| 8 | *D8Mit249* | 37 | 10 | 12 | 9 | 13 | 47.7 ± 7.5 | 0.091 |
| 8 | *D8Mit121* | 67 | 12 | 10 | 9 | 13 | 43.2 ± 7.5 | 0.818 |
| 9 | *D9Mit191* | 26 | 10 | 12 | 10 | 12 | 50 ± 7.5 | 0.000 |
| 9 | *D9Mit182* | 55 | 8 | 14 | 12 | 10 | 59.1 ± 7.4 | 1.455 |
| 10 | *D10Mit170* | 29 | 21 | 1 | 2 | 20 | 6.8 ± 3.7 | 32.818 |
| 10 | *D10Mit95* | 51 | 14 | 8 | 6 | 16 | 31.8 ± 7.0 | 5.818 |
| **11** | ***D11Mit236*** | **20** | **13** | **9** | **8** | **14** | **38.6 ± 7.3** | **2.273** |
| 11 | *D11Mit212* | 50 | 9 | 13 | 12 | 10 | 56.8 ± 7.5 | 0.818 |
| 12 | *D12Mit201* | 29 | 12 | 10 | 12 | 10 | 50 ± 7.5 | 0.000 |
| 12 | *D12Mit28* | 52 | 11 | 11 | 12 | 10 | 52.3 ± 7.5 | 0.091 |
| 13 | *D13Mit221* | 30 | 9 | 13 | 11 | 11 | 54.5 ± 7.5 | 0.364 |
| 13 | *D13Mit213* | 55 | 14 | 8 | 10 | 12 | 40.9 ± 7.4 | 1.455 |
| **14** | ***D14Mit203*** | **28.3** | **7** | **15** | **15** | **7** | **68.2 ± 7.0** | **5.818** |
| **14** | ***D14Mit195*** | **44.3** | **9** | **13** | **14** | **8** | **61.4 ± 7.3** | **2.272** |
| 15 | *D15Mit63* | 29.2 | 12 | 10 | 7 | 15 | 38.6 ± 7.3 | 2.27 |
| **15** | ***D15Mit159*** | **49.6** | **10** | **12** | **4** | **18** | **36.4 ± 7.3** | **3.27** |
| 16 | *D16Mit4* | 27.3 | 13 | 9 | 9 | 13 | 40.9 ± 7.4 | 1.455 |
| 16 | *D16Mit114* | 44.5 | 12 | 10 | 13 | 9 | 52.3 ± 7.5 | 0.091 |
| 17 | *D17Mit152* | 37.7 | 14 | 8 | 13 | 9 | 47.7 ± 7.5 | 0.091 |
| 17 | *D17Mit221* | 56.7 | 13 | 9 | 16 | 6 | 56.8 ± 7.5 | 0.818 |
| 18 | *D18Mit60* | 16 | 14 | 8 | 10 | 12 | 40.9 ± 7.4 | 1.455 |
| 18 | *D18Mit40* | 37 | 13 | 9 | 9 | 13 | 40.9 ± 7.4 | 1.455 |
| 19 | *D19Mit61* | 9 | 11 | 11 | 10 | 12 | 47.7 ± 7.5 | 0.091 |
| 19 | *D19Mit91* | 47 | 9 | 13 | 11 | 11 | 54.5 ± 7.5 | 0.364 |

[a]Chromosome.
[b]Affected homo and normal hetero represent parental combination.
[c]Affected hetero and normal homo represent recombinant combination.

We finally selected 38 from 75 markers for secondary screening after checking polymorphism between BALB/c and C57BL/6J. Their positions of 38 markers from each 19 chromosomes are shown in Table 2. Four more markers on chromosome 10 are described in Table 3.

***Statistic analysis.*** The BMD data was assessed using student's *t* - test (GraphPad Instate, V2.05a). The

**Table 3.** Further linkage analysis of causative gene on chromosome 10 for SBS mice

| Markers | Position (cM) | Affected | | Normal | | Recombination value (%) | $X^2$ analysis |
|---|---|---|---|---|---|---|---|
| | | Homo[a] | Hetero[b] | Homo[b] | Hetero[a] | | |
| *D10Mit248* | 7 | 67 | 5 | 4 | 66 | 6.3 ± 2.0 | 108.282[c] |
| *D10Mit51* | 9 | 69 | 3 | 3 | 67 | 4.2 ± 1.7 | 119.014[c] |
| ***sbs*** | | **72** | **0** | **0** | **70** | **0 ± 0** | |
| *D10Mit283* | 16 | 72 | 0 | 1 | 69 | 0.7 ± 0.7 | 138.028[c] |
| *D10Mit106* | 17 | 71 | 1 | 2 | 68 | 2.1 ± 1.2 | 130.253[c] |
| *D10Mit170* | 29 | 64 | 8 | 11 | 59 | 13.4 ± 2.9 | 76.169[c] |

[a]Affected homo and normal hetero represent parental combination.
[b]Affected hetero and normal homo represent recombinant combination.
[c]$P < .01$, difference is very significant.

genotypic difference of *SSLPs* was assessed by recombination values and $X^2$ analysis, which calculated according to recombination value = (RC/PC + RC) × 100% and the formula $X^2 = (PC - RC)^2/N$, where PC = parental combinations, RC = recombinant combinations, and N = the total number of offspring scored. The standard error (SE) of the recombination value was calculated as $SE = [p(1 - p)/N]^{1/2}$, where *P* is the recombination value.

## RESULTS

***Body weight changes.*** SBS and normal mouse are indistinguishable macroscopically in size at birth (data not shown). At 1 week of age, their difference of body size was just apparent (Fig. 1A). However, the onset of body weight changes of SBS mouse was detected from birth through body weight measured. As compared with normal mice, the body weight mean of normal mice was 1.54 ± 0.177 g, whereas the body weight mean of SBS mice was 1.37 ± 0.085 g at birth (Fig. 3A). Body weight changes were significant between SBS and normal mice from birth to 14 weeks of age. Body weight difference between them has widened from birth to 3 weeks of age. At 3 weeks of age, the body weight mean of normal mice was 11.20 ± 1.634 g, and the body weight mean of SBS mice was 4.92 ± 1.299 g. That of SBS mice was approximately 43.7% the body weight mean of normal mice and the difference was most significant (Fig. 3B). Body weight difference between them was gradually reduced from 3 to 14 weeks of age ($P < 0.001$ or $< 0.01$).

***Segregation of $N_2$ backcross progeny.*** The segregation results of the phenotypes in the $N_2$ backcross progeny are represented in Table 1. Total 142 $N_2$ backcross progeny were produced resulting from mating 4 SBS males to 8 (SBS × C57BL/6J) $F_1$ females hybrids. All [(SBS × C57BL/6J) $F_1$ × SBS] $N_2$ hybrids were observed to be phenotypical normal. Seventy of 142 $N_2$ backcross mice were not affected. The incidence of dwarfism in $N_2$ backcross progeny was 50.7%. There were no sex differences in $N_2$ backcross progeny.

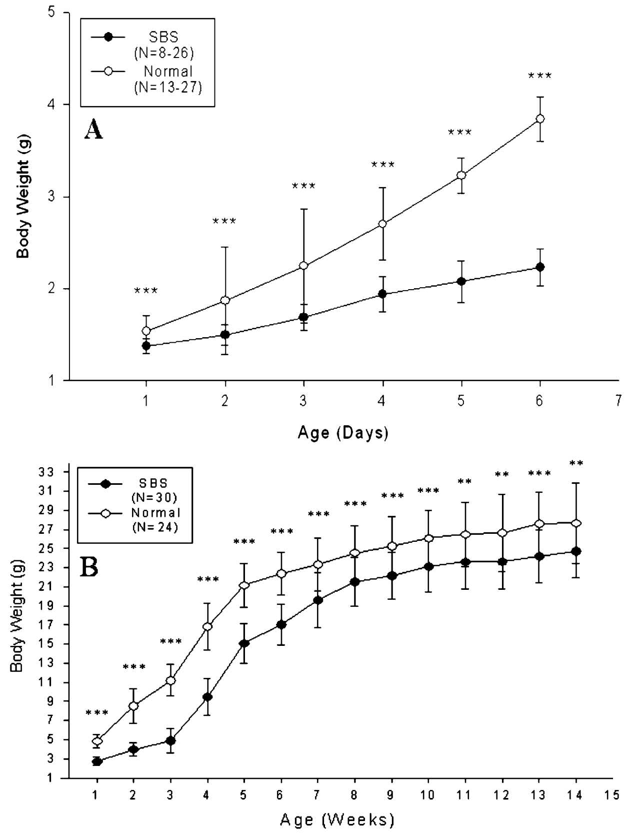

**Fig. 3.** Growth curve of affected and normal mice from birth to 6 days of age (A) and from 1 to 14 weeks of age (B), respectively [$^{**}P < 0.01$, $^{**}P < 0.001$ (student's *t*-test) VS normal mice].

***BMD changes.*** Difference was found in mean forearm BMD, femur BMD, and total body BMD at 3 and 6 weeks of age between the SBS and normal mice. As compared with the normal mice at 3 weeks of age, the

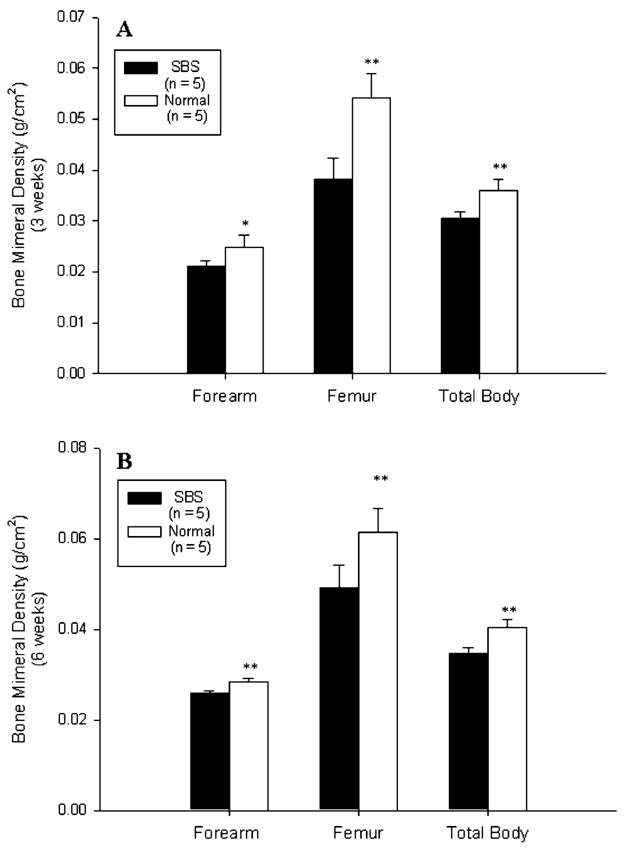

**Fig. 4.** Comparison of BMD at forearm, femur, and total body between SBS mice (n = 5) and normal mice (n = 5) at 3 (A) and 6 weeks of age (B), respectively. The BMD (forearm, femur, and total body) levels in SBS mice was shown to be significantly lower than in normal mice [* $p < 0.05$, ** $p < 0.01$ (student's *t*-test) VS normal mice].

mean BMD was 15.3%, 29.7% and 15.5% lower at forearm, femur, and total body in the SBS mice (Fig. 4A). At 6 weeks of age, the mean BMD at above same positions in SBS mice was 8.7%, 20.1% and 14.1% lower than in normal mice (Fig. 4B).

***Genetic mapping.*** In this study, $N_2$ mice were used for linkage analysis. Affected 22 $N_2$ mice and 22 normal mice were initially genotyped with 37 microsatellite markers on all 19 autosomal chromosomes, with an average interval of approximately 30 centimorgen (cM). However, the result of genotyping was not visible (data not shown). 38 new microsatellite markers were collected once again for secondary screen on all 19 autosomal chromosomes (Table 2). By linkage analysis of the causative gene in $N_2$ backcross mice, a strong linkage was expected on mouse chromosome 10 around *D10Mit170*. Further linkage analysis was narrowed to chromosome 10 with additional 4 microsatellite markers, *D10Mit248*, *D10Mit51*, *D10Mit283*, and *D10Mit106*. A total of 142 $N_2$ backcross mice consisted of 72 affected mice and 70 normal mice were genotyped for linkage analysis. Table 3 represents the genotypes of all $N_2$ backcross mice for 5 microsatellite markers on chromosome 10. All affected $N_2$ backcross mice showed dwarfism had the BALB/c homozygote genotypes at *D10Mit283* on chromosome 10, and all normal $N_2$ backcross mice had the BALB/c × C57BL/6J heterozygote genotypes at *D10Mit283* on chromosome 10 except for only one normal $N_2$ backcross mouse. The one normal $N_2$ backcross mouse had the BALB/c homozygote genotype at *D10Mit283*. Fig. 5 shows the distribution of haplotypes.

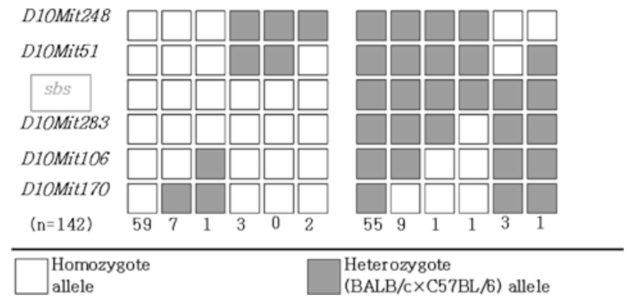

**Fig. 5.** Linkage analysis of the causative gene localized on chromosome 10. A total of 142 $N_2$ progeny, 72 affected and 70 normal mice were genotyped. Distribution of the haplotypes for chromosome 10 was in affected and normal mice. White boxes represent homozygote allele (C/C) genotype, and grey boxes represent heterozygote allele (B/C) genotype at each locus. The microsatellite markers were shown on the left.

## DISCUSSION

Syndromes of heritable dwarfism in humans and animals can be caused by states of a variety of complication and genetic heterogeneity, with either dominant or recessive inheritance, some of which are related to point mutations. Using random ENU-mutagenesis, we generated novel mouse model for dwarfism which was named SBS mouse. The SBS mouse exhibit heritable dwarfism in a recessive Mendelian pattern (Cho *et al.*, 2003). In this study, we examined body weight changes and bone mineral density (BMD), and we also carried out genetic linkage analysis to map the causative gene of SBS mouse developed by ENU mutagenesis.

The phenotypic differences in body size manifest from 1 to 14 weeks of age during puberty except birth, but body weight changes appear to be already present at birth (Fig. 3). Measurements of newborn SBS mice yielded body weights of $1.3 \pm 0.08$ g, comparable with normal mice 1.5 ± 0.18 g from similar litter sizes (data not shown).

In results of bone density analysis, BMD of the fore-arm, femur, and total body in SBS mice were significantly low compared with normal mice (Fig. 4). BMD has been employed most commonly as the index for defining and studying osteoporosis (Deng and Recker, 2004; Rosen *et al.*, 2001). The World Health Organization (WHO) defines osteoporosis as a value for BMD or bone mineral content (BMC) that is more than 2.5 SD below the young gender and adult mean value (WHO, 1994). As such extensive molecular genetic studies have been conducted to search for genes underlying BMD variation (Duncan *et al.*, 1999; Braga *et al.*, 2002; Deng *et al.*, 2002; Wynne *et al.*, 2002). Osteoporosis and its associated phenotypes, which showed bone ache, body size shorten and bone fracture, are under the strong genetic control (Hwang *et al.*, 2003). Identification and characterization of specific loci or genes involved in determining osteoporosis and its associated phenotypes will contribute to a greater understanding of the pathogenesis of osteoporosis, and ultimately might lead to the development of better diagnosis, prevention and treatment strategies (Hwang *et al.*, 2003). A promising approach is to map quantitative traits in experimental animal models and then search syntenic regions of the human genome for genes defining these traits in human. The genetics of osteoporosis and associated traits have been studied extensively in inbred strains of experimental animals (Li *et al.*, 2002; Klein *et al.*, 1998, 2001; Shimizu *et al.*, 1999, 2001; Beamer *et al.*, 1999, 2001; Drake *et al.*, 2001; Benes *et al.*, 2000; Masinde *et al.*, 2002). Huang *et al.* reported the Quantitative Traits Linkage (QTL) mapping results for BMD in experimental animals and the associated human homologous regions are summarized. The QTLs of osteoporosis have been mapped in all 19 chromosomes of experimental animals except chromosome 10 (Hwang *et al.*, 2003).

From this study, we found that the causative gene (*sbs*) was mapped on chromosome 10. The map order and the distance between markers were *D10Mit248* - 2.1 cM - *D10Mit51* - 4.2 cM - ***sbs*** - 0.7 cM - *D10Mit283* - 1.4 cM - *D10Mit106* - 11.2 cM - *D10Mit170* (Fig. 6) (Dietrich *et al.*, 1996). These results showed that *D10Mit51* and *D10Mit283* provide a useful anchor for the isolation of the *sbs* gene. We could not map the *sbs* locus on a more precise position as no additional effective marker loci between these loci were obtained. Comparative maps of genetic, Jackson Laboratory and sequence indicate chromosomal region in which synteny is conserved (Fig. 5, 6). In this regions, possible

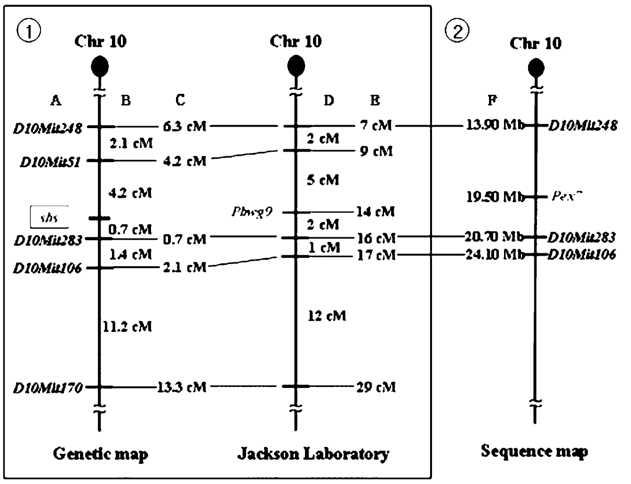

**Fig. 6.** A partial genetic ① and sequence map ② (http://ensembl.org/Mus_musculus/contigview?geneid=NM_008822) of chromosome 10 compared with distance of microsatellite markers from Jackson Laboratory, and the location of *Pbwag9* and *Pex7* gene: microsatellite markers (A), distance between markers (B), distance from causative gene (C, *sbs*), distance between markers from MGI (D), distance from centromere from MGI (E) and microsatellite markers sequence size (F) were presented in the map.

candidate genes for dwarfism phenotypes, including postnatal body weight growth 9 (*Pbwg9*) and peroxisome biogenesis factor 7 (*Pex7*) have been mapped on mouse chromosome 10 (Brites *et al.*, 2003; Ishikawa and Namikawa, 2004).

Postnatal body weight growth 9 (*Pbwg9*) was located by QTL mapping whose location is 14 cM from centromere on chromosome 10, which mainly regulated the function in the middle growth phase of entire growth process (Ishikawa and Namikawa 2004; Cheverud *et al.*, 1996). Therefore, The *Pbwg9* gene could be a candidate gene for *sbs* gene. Mutations in the peroxisome biogenesis factor (*Pex7*) encoding the receptor for a class of peroxisomal matrix enzymes have been found in patients of rhizomelic chorndrodysplasia punctata (*RCDP*), who show many abnormal phenotype such as dwarfism, abnormal lipid chemistry, abnormal cartilage development, reduced bone density and cataracts (Brites *et al.*, 2003; Braverman *et al.*, 1997; Purdue *et al.*, 1997). The *Pex7* gene is considered as a strong candidate gene for *sbs* gene. The mapping of these candidate genes in the region on the chromosome 10 and the identification of markers loci tightly linked to the *sbs* locus will reveal correspondence between these genes and the dwarfism. For further studies, it may require identifying the gene mutations that occurred and demonstrate the features of the mutations by sequence analysis.

## CONCLUSION

Body weight changes were elucidated in detail between affected mice and normal mice, which were examined by two time phases; one is once one day from birth to 6 days of age and another is from 1 to 14 weeks of age at intervals of one week. The difference of body weight, which was very significant, was verified.

Inheritance mode of the mouse was further confirmed through mating experiment in this study. There was no difference of the number between male and female, but the number of normal and affected animals showed segregation ration of 1 : 1 in backcross progeny. It was determined that mutant gene feature is autosomal single recessive inheritance based on Mendelian law.

Difference of bone mineral density (BMD) between normal and affected mice at 3 and 6 weeks of age during puberty is very notable. Thus, we expect SBS mice might be a good model for the study of osteoporosis related with BMD for studies in the future.

For genetic study, we narrowed down the genetic map position between *D10Mit51* and *D10Mit283*, and demonstrated the possibility of postnatal body weight growth 9 (*Pbwg9*) and the peroxisome biogenesis factor (*Pex7*) as candidate gene for *sbs*.

This study has not been able to entirely elucidate every phenotype of SBS mouse, but the biochemical and physiological analysis and fine mapping for positional cloning of the mutant gene will be in progress.

## ACKNOWLEDGEMENT

This study was supported by National Research Laboratory Program (2000-N-NL-01-c-207) from the Ministry of Science and Technology of Korea.

## References

[CR1] Argentin G, Cicchetti R (2000). *In vitro* proliferation of achondroplastic and normal mouse chondrocytes, before and after basic fibroblast growth factor stimulation. Cell Prolif..

[CR2] Beamer WG, Shultz KL, Churchill GA, Donahue LR (1999). Quantitative trait loci for bone density in C57BL/6J and CAST/EiJ inbred mice. Mamm. Genome..

[CR3] Beamer WG, Shultz KL, Donahue LR, Rosen CJ (2001). Quantitative trait loci for femoral and lumbar vertebral bone mineral density in C57BL/6J and C3H/HeJ inbred strains of mice. J. Bone Miner. Res..

[CR4] Benes H, Weinstein RS, Zheng W, Thaden JJ, Shmookler RRJ (2000). Chromosomal mapping of osteopenia-associated quantitative trait loci using closely related mouse strains. J. Bone Miner. Res..

[CR5] Braga V, Sangalli A, Malerba G, Mottes M, Mirandola S, Gatti D, Rossini M, Zamboni M, Adami S (2002). Relationship among VDR (BsmI and FokI), COLIA1, and CTR polymorphisms with bone mass, bone turnover markers, and sex hormones in men. Calcif. Tissue Int..

[CR6] Braverman N, Steel G, Obie C, Moser A, Moser H, Gould SJ, Valle D (1997). Human PEX7 encodes the peroxisomal PTS2 receptor and is responsible for rhizomelic chondrodysplasia punctata. Nat. Genet..

[CR7] Brites P, Motley AM, Gressens P, Mooyer PA, Ploegaert I, Everts V, Evrard P, Carmeliet P, Dewerchin M, Schoonjans L, Duran M, Waterham HR, Wanders RJ, Baes M (2003). Impaired neuronal migration and endochondral ossification in Pex7knockout mice: a model for rhizomelic chondrodysplasia punctata. Hum. Mol. Genet..

[CR8] Brown SDM, Hardisty RE (2003). Mutagenesis strategies for identifying novel loci associated with disease phenotypes. Semin. Cell Dev. Biol..

[CR9] Cheverud JM, Routman EJ, Duarte FA v, Swinderen B, Cothran K, Perel C (1996). Quantitative trait loci for murine growth. Genetics.

[CR10] Cho JW, Yoo JK, Cho KH, Han SS, Song CW (2003). The Devlopment of Dwarfism Mice Using ENU Mutagenesis. Kor. J. Lab. Ani. Sic..

[CR11] Davis AP, Woychik RP, Justice MJ (1999). Effective chemical mutagenesis in FVB/N mice requires low dose s of ethylnitrosourea. Mamm. Genome.

[CR12] Deng HW, Shen H, Xu FH, Deng HY, Conway T, Zhang HT, Recker RR (2002). Tests of linkage and/or association of genes for vitamin D receptor, osteocalcin, and parathyroid hormone with bone mineral density. J. Bone Miner. Res..

[CR13] Deng HW, Recker RR (2004). Gene mapping and identification for osteoporosis. J. Musculoskelet. Neuronal Interact..

[CR14] Dietrich WF, Miller J, Steen R, Merchant MA, Damron-Boles D, Husain Z, Dredge R, Daly MJ, Ingalls KA, O’Connor TJ (1996). A comprehensive genetic map of the mouse genome. Nature.

[CR15] Drake TA, Schadt E, Hannani K, Lusis AJ (2001). Genetic loci determining bone density in mice with dietinduced atherosclerosis. Physiol. Genom..

[CR16] Duncan EL, Brown MA, Sinsheimer J, Bell J, Carr AJ, Wordsworth BP, Wass JA (1999). Suggestive linkage of the parathyroid receptor type 1 to osteoporosis. J. Bone Miner. Res..

[CR17] Eason J, Hall CM, Trounce JQ (1995). Renal tubular leakage complicating microcephalic osteodysplastic primordial dwarfism. J. Med. Genet..

[CR18] Gibbs RA, Weinstock GM, Metzker ML, Muzny DM, Sodergren EJ, Scherer S, Scott G, Rat Genome Sequencing Project Consortium (2004). Genome sequence of the Brown Norway rat yields insights into mammalian evolution. Nature.

[CR19] Godfrey P, Rahal JO, Beamer WG, Copeland NG, Jenkins NA, Mayo KE (1993). GHRH receptor of little mice contains a missense mutation in the extracellular domain that disrupts receptor function. Nat. Genet..

[CR20] Hitotsumachi S, Carperter DA, Russel WL (1985). Dose repetition increases the mutagenic effectiveness of *N*-ethyl-*N*-nitrosourea in mouse spermatogonia. Proc. Matl. Acad. Sci..

[CR21] Hrabe de Angelis MH, Flaswinkel H, Fuchs H, Balling R, Balling R (2000). Genome-wide, large-scale production of mutant mice by ENU mutagenesis. Nat. Genet..

[CR22] Hu CT, O’shaughnessy KM (2001). Glycerol-enhanced mini-polyacrylamide gel electrophoresis for the separation of differentially expressed DNA fragments in cDNA representational difference analysis. Electrophoresis.

[CR23] Hwang QY, Recker RR, Deng HW (2003). Searching for osteoporosis genes in the post-genome era: progress and challenges. Osteoporos. Int..

[CR24] Ishikawa A, Namikawa T (2004). Mapping major quantitative trait loci for postnatal growth in an intersubspecific backcross between C57BL/6J and Philippine wild mice by using principal component analysis. Genes Genet. Syst..

[CR25] Jansson JO, Downs TR, Beamer WG, Frohman LA (1986). Receptor-associated resistance to growth hormonereleasing factor in dwarf “little” mice. Science.

[CR26] Justice MJ, Bode VC (1986). Induction of new mutations in a mouse t-haplotype using ethylnitrosourea mutagenesis. Gene Res..

[CR27] Justice MJ, Noveroske JK, Weber JS, Zheng B, Bradley A (1999). Mouse ENU mutagenesis. Hum. Mol. Genetics.

[CR28] Justice MJ, Carpenter DA, Favor J, Neuhauser KA, Hrabe de Angelis M, Soewarto D, Bode VC (2000). Effects of ENU doseage on mouse strains. Mamm. Genome.

[CR29] Kim JC, Shin DH, Kim SH, Yang YS, Oh KS, Jiang CZ, Chung MK (2006). Teratogenicity evaluation of 2-bromopropane using rat whole embryo culture. J. Toxicol. Pub. Health.

[CR30] Klein RF, Mitchell SR, Phillips TJ, Belknap JK, Orwoll ES (1998). Quantitative trait loci affecting peak bone mineral density in mice. J. Bone Miner. Res..

[CR31] Klein RF, Carlos AS, Vartanian KA, Chambers VK, Orwoll ES (2001). Confirmation and fine mapping of chromosomal regions influencing peak bone mass in mice. J. Bone Miner. Res..

[CR32] Kwack SJ, Cho DH (2005). The recommended approaches and recent trends in reproductive and developmental toxicology. J. Toxicol. Pub. Health.

[CR33] Lander ES, Linton LM, Birren B, Nusbaum C, Zody MC, Baldwin J, Devon K, Dewar K, Wyman D (2001). Initial sequencing and analysis of the human genome. Nature.

[CR34] Li S C, Crenshaw EB, Rawson EJ, Simmonds DM, Swanson LV, Rosenfeld MG (1990). Dwarf locus mutants lacking three pituitary cell types result from mutations in the POU-domain gene pit-1. Nature.

[CR35] Li X, Masinde G, Gu W, Wergedal J, Mohan S, Baylink DJ (2002). Genetic dissection of femur breaking strength in a large population (MRL/MpJ × SJL/J) of F2 mice: single QTL effects, epistasis, and pleiotropy. Genomics.

[CR36] Lin SC, Lin CR, Gukovsky I, Lusis AJ, Sawchenco PE, Rosenfeld MG (1993). Molecular basis of the little mouse phenotype and implications for cell-type specific growth. Nature.

[CR37] Lira SA, Kalla KA, Glass CK, Drolet DW, Rosenfeld MG (1993). Synergistic interactions between Pit-1 and other elements are required for effective somatotroph rat growth hormone gene expression in transgenic mice. Mol. Endocrinol..

[CR38] Masinde GL, Li X, Gu W, Wergedal J, Mohan S, Baylink DJ (2002). Quantitative trait loci for bone density in mice: the genes determining total skeletal density and femur density show little overlap in F2 mice. Calcif. Tissue Int..

[CR39] Meyer CW, Korthaus D, Jagla W, Cornali E, Grosse J, Fuchs H, Klingenspor M, Roemheld S, Tschop M, Heldmaier G, De Angelis MH, Nehls M (2004). A novel missense mutation in the mouse growth hormone gene causes semidominant dwarfism, hyperghrelinemia, and obesity. Endocrinology.

[CR40] Noveroske JK, Weber JS, Justice MJ (2000). The mutagenic action of ENU in the mouse. Mamm. Genome.

[CR41] Purchase IFH (2001). Assessment of the risk of exposure to chemical carcinogens. J. Toxicol. Pub. Health.

[CR42] Purdue PE, Zhang JW, Skoneczny M, Lazarow PB (1997). Rhizomelic chondrodysplasia punctata is caused by deficiency of human PEX7, a homologue of the yeast PTS2 receptor. Nat. Genet..

[CR43] Russel WL, Kelly EM, Hunsicker PR, Bangham JW, Maddux SC, Phipps EL (1979). Specific-locus test shows ethylnitrosourea to be the most potent mutagen in the mouse. Proc. Matl. Acad. Sci..

[CR44] Russel WL, Hunsicker PR, Carpenter DA, Cornett CV, Guinn GM (1982). Effect of dose fractionation on the ethylnitrosourea induction of specificlocus mutations in mouse spermatogonia. Proc. Matl. Acad. Sci..

[CR45] Russel WL, Hunsicker PR, Raymer GD, Steele MH, Stelzmer KF, Thompson HM (1982). Doseresponse curve for the ethylnitrosourea-inducted specificlocus mutations in mouse spermatogonia. Proc. Matl. Acad. Sci..

[CR46] Ressell LB, Russell WL (1992). Frequency and nature of specific-locus mutations induced in female mice by radiations and chemicals: a review. Mutat. Res..

[CR47] Rosen CJ, Beamer WG, Donahue LR (2001). Defining the genetics of osteoporosis: Using the mouse to understand man. Osteoporos. Int..

[CR48] Shelovsky A, McDonald JD, Symula DM, Dove WF (1993). Mouse models of human phenylketonuria. Genetics.

[CR49] Shibayama K, Ohyama Y, Ono M, Furudate S (1993). Expression of mRNA coding for pituitary hormones and pituitary-specific transcription factor in the pituitary gland of the rdw rat with hereditary dwarfism. J. Endocrinol..

[CR50] Shimizu M, Higuchi K, Bennett B, Xia C, Hosokawa M (1999). Identification of peak bone mass QTL in spontaneously osteoporotic mouse strain. Mamm. Genome.

[CR51] Shimizu M, Higuchi K, Kasai S, Nakamura T, Hosokawa M (2001). Chromosome 13 locus, Pbd2, regulates bone density in mice. J. Bone Miner. Res..

[CR52] Soewarto D, Fella C, Teubner A, Rathkolb B, Pargent W, Heffner S, Angelis MH (2000). The large-scale Munich ENU-mouse-mutagenesis screen. Mamm. Genome.

[CR53] Sornson MW, Wu W, Dasen JS (1996). Pituitary lineage determination by the Prophet of Pit-1 homeodomain factor defective in Ames dwarfism. Nature.

[CR54] Tsuji T, Kunieda T (2005). A loss-of-function mutation in natriuretic peptide receptor 2 (Npr2) gene is responsible for disproportionate dwarfism in cn/cn mouse. J. Biol. Chem..

[CR55] Takeuchi T, Suzuki H, Sakurai S, Nogami H, Okuma S, Ishikawa H (1990). Molecular mechanism of growth hormone (GH) deficiency in the spontaneous dwarf rat: detection of abnormal splicing of GH messenger ribonucleic acid by polymerase chain reaction. Endocrinology.

[CR56] Wang Y, Spatz MK, Kannan K, Hayk H, Avivi A, Gorivodsky M, Pines M, Yayon A, Lonai P, Givol D (1999). A mouse model for achondroplasia produced by targeting fibroblast growth factor receptor 3. Proc. Natl. Acad. Sci..

[CR57] Waterston RH, Lindblad-Toh K, Birney E, Rogers J, Abril JF, Agarwal P, Agarwala R, Mouse Genome sequencing Consortium (2002). Initial sequencing and comparative analysis of the mouse genome. Nature.

[CR58] WHO (1994). Assessment of fracture risk and its application to screening for postmenopausal osteoporosis. World Health Organ Tech. Report Series No..

[CR59] Wynne F, Drummond F, O’Sullivan K, Daly M, Shanahan F, Molloy MG, Quane KA (2002). Investigation of the genetic influence of the OPG, VDR (Fok1), and COLIA1 Sp1 polymorphisms on BMD in the Irish population. Calcif. Tissue Int..

[CR60] Zhou X, Benson KF, Ashar HR, Chada K (1995). Mutation responsible for the mouse pygmy phenotype in the developmentally regulated factor HMGI-C. Nature.

